# Organic Matter Regulates Ammonia-Oxidizing Bacterial and Archaeal Communities in the Surface Sediments of *Ctenopharyngodon idellus* Aquaculture Ponds

**DOI:** 10.3389/fmicb.2018.02290

**Published:** 2018-09-24

**Authors:** Lili Dai, Chengqing Liu, Liqin Yu, Chaofeng Song, Liang Peng, Xiaoli Li, Ling Tao, Gu Li

**Affiliations:** ^1^Key Laboratory of Freshwater Biodiversity Conservation, Ministry of Agriculture, Yangtze River Fisheries Research Institute, Chinese Academy of Fishery Sciences, Wuhan, China; ^2^College of Fisheries, Huazhong Agricultural University, Wuhan, China; ^3^College of Marine Sciences, Shanghai Ocean University, Shanghai, China

**Keywords:** ammonia-oxidizing bacteria, ammonia-oxidizing archaea, *Ctenopharyngodon idellus*, aquaculture pond, sediment, organic matter

## Abstract

Ammonia-oxidizing bacteria (AOB) and archaea (AOA) play important roles in nitrogen removal in aquaculture ponds, but their distribution and the environmental factors that drive their distribution are largely unknown. In this study, we collected surface sediment samples from *Ctenopharyngodon idellus* ponds in three different areas in China that practice aquaculture. The community structure of AOB and AOA and physicochemical characteristics in the ponds were investigated. The results showed that AOA were more abundant than AOB in all sampling ponds except one, but sediment AOB and AOA numbers varied greatly between ponds. Correlation analyses indicated a significant correlation between the abundance of AOB and arylsulfatase, as well as the abundance of AOA and total nitrogen (TN) and arylsulfatase. In addition, AOB/AOA ratio was found to be significantly correlated with the microbial biomass carbon. AOB were grouped into seven clusters affiliated to *Nitrosospira* and *Nitrosomonas*, and AOA were grouped into six clusters affiliated to *Nitrososphaera*, *Nitrososphaera* sister group, and *Nitrosopumilus*. AOB/AOA diversity in the surface sediments of aquaculture ponds varied according to the levels of total organic carbon (TOC), and AOB and AOA diversity was significantly correlated with arylsulfatase and β-glucosidase, respectively. The compositions of the AOB communities were also found to be significantly influenced by sediment eutrophic status (TOC and TN levels), and pH. In addition, concentrations of acid phosphatase and arylsulfatase in surface sediments were significantly correlated with the prominent bacterial *amoA* genotypes, and concentrations of TOC and urease were found to be significantly correlated with the prominent archaeal *amoA* genotype compositions. Taken together, our results indicated that AOB and AOA communities in the surface sediments of *Ctenopharyngodon idellus* aquaculture ponds are regulated by organic matter and its availability to the microorganisms.

## Introduction

Globally, aquaculture has been continuously growing in the past 50 years to around 73.8 million tons (not including aquatic plants) in 2014 (FAO, 2016). China has played a major role in its growth and represents more than 60 percent of the global aquaculture production (FAO, 2016). According to the Bureau of Fisheries, 31.79 million tons of freshwater aquaculture products were generated in China in 2016, with an estimated value of US$91.51 billion (Bureau of Fisheries, 2017). However, along with these remarkable achievements, concerns have been raised about the negative environmental effects of increasing productivity which is largely based on high-density pond cultivation. Fertilizers and supplementary feedstuffs are largely used in intensive aquaculture to achieve higher productivity, resulting in the accumulation of nitrogen in pond sediments owing to both the excrements of the species cultivated and excessive residual matter (Avnimelech and Ritvo, 2003). Excessive nitrogen in sediments not only spoils the aquaculture environment and leads to the death of the cultivated species (Camargo and Alonso, 2006), but also affects the surrounding water bodies when discharged (Cao et al., 2007). Thus, the removal of excess nitrogen from the pond systems of high-density aquaculture is of great importance.

Nitrification is considered an essential process during nitrogen loss in ecosystems (Wu et al., 2017). This process consist of two steps, where ammonia is first oxidized to nitrite and subsequently to nitrate, which are thought to be implemented either by separate groups of microorganisms (Vlaeminck et al., 2011) or by a single group of microorganisms, the latter only having been discovered in recent years (known as “comammox”; van Kessel et al., 2015). Microbial ammonia oxidation is the first and rate-limiting step of the two-step nitrification, and has been intensively investigated. Ammonia oxidation is known to be mediated by both ammonia-oxidizing bacteria (AOB) and archaea (AOA) (Venter et al., 2004; Treusch et al., 2005). Terrestrial AOB are classified into two genera *Nitrosospira* and *Nitrosomonas* based on the gene sequences of 16S rRNA (Head et al., 1993; Teske et al., 1994) and ammonia monooxygenase (*amoA*) (Rotthauwe et al., 1997; Purkhold et al., 2000). AOA were only identified in recent years but were found to be ubiquitous. AOA consist of *Nitrosopumilus* cluster (group I.1a), *Nitrosotalea* cluster (group I.1a-associated), *Nitrososphaera* cluster (group I.1b), *Nitrososphaera* sister cluster, and *Nitrosocaldus* cluster (ThAOA) (Pester et al., 2012). AOB and AOA have been found to be common in various environments, including estuaries (Beman and Francis, 2006; Sahan and Muyzer, 2008), soils (Leininger et al., 2006), rivers (Sun et al., 2013), lakes (Liu et al., 2015), reservoirs (Wang X. et al., 2014), and wetlands (Yang et al., 2014). The distribution of AOB and AOA varied according to the different habitats being studied, and responded to different environmental factors. For instance, pH, salinity, and nutrients could affect AOB community composition (Li et al., 2015), while water depth, redox variation, and concentration of ammonium drive AOA distribution (Flood et al., 2015). Although their wide distribution and high abundance have been reported (Lu et al., 2016), our current understanding of the distribution characteristics of AOB and AOA in aquaculture ponds is still very limited.

Aquaculture ponds are unique anthropogenic aquatic ecosystems characterized by regular nutrient input. Incomplete assimilation by cultivated species cause continuous accumulation of nutrients in pond sediments, mainly in the form of organic matter (Jimenez-Montealegre et al., 2005). With the decomposition of organic matter, oxygen is depleted and large quantities of ammonia accumulate in the sediment layer (Hargreaves, 1998). The overnutrition and the highly reduced environment in aquaculture ponds can cause significant effect on the nitrification process and may tend to favor specific microbial communities. Although the diversity and abundance of pond sediment AOB and AOA have been characterized in previous studies (Lu et al., 2015, 2016; Zhou et al., 2017), these studies were conducted in single pond systems, and the distribution characteristics of AOB and AOA in aquaculture pond sediments, as well as the driving factors of this distribution, are still unclear.

In this study, we collected surface sediment samples from *Ctenopharyngodon idellus* aquaculture ponds located in three different areas: Heilongjiang, Changjiang, and Zhujiang; these are considered to be the leading freshwater aquaculture-producing areas in North and South China (Wang et al., 2015). By comparing the AOB and AOA communities and the environmental factors between the different sampling areas, as well as correspondingly analyzing the microorganisms and the factors, the present study aims to characterize the distribution of AOB and AOA in aquaculture pond sediments, and determine possible driving environmental factors.

## Materials and Methods

### Site Description and Sample Collection

Water and sediment samples were collected from *Ctenopharyngodon idellus* aquaculture ponds located in three different areas in China: Changjiang (30°18′34′′-30°18′45′′N, 112°03′53′′-112°04′17′′E), Heilongjiang (46°23′32′′-46°23′50′′N, 126°44′25′′-126°44′42′′E), and Zhujiang (22°36′33′′-22°36′37′′N, 113°30′49′′-113°30′58′′E) aquaculture areas. Three ponds were sampled in each area. The sampling ponds had a surface area of ∼7000–27000 m^2^ and a depth of ∼1.5–3.5 m. In the sampled ponds, *Ctenopharyngodon idellus* intercropped with *Hypophthalmichthys molitrix*, and *Hypophthalmichthys nobilis* were raised for commercial use. Detailed information of *Ctenopharyngodon idellus* culturing is listed in **Supplementary Table S1**.

Surface sediment samples (0–5 cm) were collected in triplicate from the aquaculture ponds between May and June 2017 using a core sampler. In each pond, the sediments were mixed and placed in sterile plastic bags, then sealed and transported to the laboratory on ice. One portion was used for the determination of ammonia oxidation activities immediately after arrival, and another portion was used for the analysis of physicochemical characteristics. Subsamples were stored at -80°C for subsequent DNA extractions and molecular analysis. Water samples were also collected for characterization. Water temperature, dissolved oxygen (DO), and pH were recorded *in situ* using a handheld smarTROLL Multiparameter (In-Situ Inc., United States).

### Physicochemical Analysis

For the water samples, ammonium (NH_4_^+^), nitrite (NO_2_^-^), nitrate (NO_3_^-^), total nitrogen (TN), and total phosphorus (TP) were determined according to standard protocols (China Environmental Protection Agency, 2002). Sediment pH was determined by mixing the sample thoroughly with water in a ratio of 1:2.5 (m/v). NH_4_^+^, NO_2_^-^ and NO_3_^-^ were extracted from the sediment with 1 mol/L KCl solution and measured by spectrophotometric methods (Lu et al., 2016). The TN and TP of the sediment were measured using the semi-micro Kjeldahl method and the alkali fusion - Mo-Sb anti spectrophotometric method, respectively. Sediment organic carbon (TOC) content was determined by using the potassium dichromate oxidation spectrophotometric method (Song et al., 2012). The concentrations of urea were measured according to the method of Therkildsen et al. (1996), with small modifications. Briefly, urea was extracted from ∼35 g of sediment with 15 ml of 1M KCl. The slurry was centrifuged to terminate the extraction after 10 min. Urea concentrations in the supernatant were then measured using the diacetylmonoxime method (Price and Harrison, 1987). The concentrations of water- and acid-soluble sulfate in the sediment were determined by the gravimetric method (American Society for Testing and Materials, 1973).

### Microbial Biomass and Soluble Carbon

Microbial biomass carbon (MBC) in the sediment was determined using the fumigation-extraction method (Vance et al., 1987). Briefly, fresh sediments were incubated with chloroform for 24 h, then extracted with 0.5 mol/L potassium sulfate solution, and the difference of the carbon content between the fumigated and the unfumigated samples was determined to be the carbon content of microorganisms in the sediments. The non-fumigated fraction was directly used as the dissolved organic carbon (DOC).

### Sediment Enzymes

The activities of four enzymes indicative of C-cycling (β-galactosidase), N-cycling (urease), P-cycling (acid phosphatase) and S-cycling (arylsulfatase) were evaluated with 4-nitrophenyl beta-D-glucopyranoside, urea, 4-nitrophenyl phosphate disodium, and 4-nitrophenyl sulfate potassium as the substrates, respectively, according to Bowles et al. (2014). Briefly, the enzyme activities were assayed using 1 g of air-dried soil which was incubated with the substrate for 1 h or 24 h (37°C) at the optimal pH. Enzyme activities were assayed in duplicate with one control, to which the substrate was added after incubation and subtracted from the sample value. Air-dried sediments were used for enzyme analysis as previous studies have indicated that no differences were detected between assays with air-dried and field-moist samples, or resulted in the same ranking of treatments between field-moist and air-dried samples for the enzymes we evaluated (e.g., Bandick and Dick, 1999; Li and Sarah, 2003).

### Potential Ammonia Oxidation Rates (PNRs)

The potential ammonia oxidation in sediments was determined by the chlorate inhibition method (Kurola et al., 2005). Briefly, sediment samples were mixed with a test medium containing 0.04 g/L KH_2_PO_4_, 0.18 g/L K_2_HPO_4_, 1.06 g/L NaClO_3_, and 0.2 g/L (NH_4_)_2_SO_4_ to form slurries. The slurries were then incubated upright in an orbital shaking incubator (175 rpm) thermostatically controlled at 25°C for 6 h. Samples of the sediment slurry were collected after 2 and 6 h of incubation, and the nitrite levels were determined. The potential ammonia oxidation rates were calculated based on the change in nitrite concentration.

### Nucleic Acid Extraction and *amoA* Gene Amplification

Approximately 1 g (wet weight) of sediment was transferred into a sterile 1.5 ml centrifuge tube. Total genomic DNA was extracted using the DNeasy^®^ PowerSoil^®^ Kit (QIAGEN, Hilden, Germany) according to the manufacturer’s instructions. Briefly, the sediment was defrosted and homogenized in lysis buffer, then subjected to disruption by bead-beating, and finally centrifuged at 10,000 *g* for 30 s. The supernatant was then added with extraction buffer and extracted twice. An additional cleaning step was conducted using the MB Spin Column. The extracted DNA was eluted in 50 μl of ultra clean water and stored at -20°C for further analyses.

The primers and reaction conditions for *amoA* gene amplification are listed in **Supplementary Table S2**. PCR amplification was performed in a 25 μl reaction volume containing 1.5 μl of DNA template (50–200 ng per reaction), 2X Utaq master mix (ZOMANBIO Inc., Beijing, China), and 1.5 μl of forward and reverse primers (10 μM).

### Quantitative Analysis by Real-Time PCR

DNA extracts were used as templates to quantify bacterial and archaeal *amoA* in the SG Fast qPCR Master Mix (High Rox) (BBI, Sangon Biotech, Shanghai, China) by real-time PCR (ABI StepOnePlus, ABI, Life Tech., Foster City, CA, United States). The primer sets used here were the same as those used in the general PCR (**Supplementary Table S2**). qPCRs were conducted in duplicate in total volumes of 20 μl containing 10 μl SybrGreen qPCR Master Mix, 2 μl template DNA (10–40 ng per reaction), and 0.4 μl of forward and reverse primers (10 μM). The reaction conditions are provided in **Supplementary Table S3**.

Inhibition by co-extracted compounds was evaluated by qPCR with different dilutions of the DNA template. Briefly, sediment DNA with 10 times dilutions was used as the template for the qPCR assay with the *amoA* primers (**Supplementary Table S2**), and the inhibition was validated by comparing the threshold cycle (Ct) values between different dilutions. The qPCR assay was performed following the same procedure described above. The results indicated that inhibition was not detected or was negligible in most cases.

Plasmids extracted from the AOB or AOA clones (see the following section) were used as a standard for the quantitative analysis of the bacterial or archaeal *amoA* gene, respectively. The plasmid concentration was determined using an UV-vis Spectrophotometer (ND-2000, Thermo Scientific, DE), and was used to calculate the copy numbers of *amoA* genes. The plasmids of the *amoA* gene clones with known concentrations were diluted to produce the standard curve over six orders of magnitude. Melting curve analysis was performed to verify the specificity of the amplification.

### Clone Library Construction and Sequencing

Clone libraries were constructed using the amplified amoA genes. PCR products were obtained and purified from size-verified gel bands using SanPrep Column PCR Product Purification kits (Sangon Biotech, Shanghai, China). The purified DNA was ligated to a pUCm-T vector (Beyotime, Shanghai, China), then transformed into Escherichia coli DH5α competent cells for colone library construction. Thirty to sixty colonies from each clone library were randomly selected. Plasmids were extracted from each colony after culturing overnight at 37°C using Uniq-10 Column Plasmid Minipreps Kits (Sangon, Shanghai, China). Plasmids of the correct size, verified by enzyme digestion and gel electrophoresis, were subjected to an ABI 3730xl DNA analyzer (Applied Biosystems, Foster City, CA, United States) for sequence analysis.

### Molecular Analysis

Chimera-free *amoA* gene sequences with similarities equal to or greater than 97% were grouped into the same operational taxonomic units (OTUs). The coverage of each clone library, the OTU-based richness and diversity indices, and the rarefaction analyses were calculated using mothur v.1.39.5 (Schloss et al., 2009). The weighted UniFrac distances between different communities were calculated using mothur. Principal Coordinates Analysis (PCoA) based on the weighted UniFrac distance were performed using the ape package (Paradis et al., 2004), and redundancy analysis (RDA) and canonical correlation analysis (CCA) were performed using the vegan package (Oksanen et al., 2018) in R v. 3.4.3 (R Core Team, 2017). Plots of heatmaps, RDA and CCA were performed using the ggplot2 (Wickham, 2009) package in R.

The bacterial and archaeal sequences recovered from clone libraries were blasted in GenBank using the BLAST^1^. The most closely related sequences and additional reference sequences were retrieved and subsequently aligned with representative clones in CLUSTALX (version 2.0.11). Phylogenetic neighbor-joining tree were constructed by MEGA (version 7.0.26).

### Statistical Analysis

All statistical analyses were performed with the IBM SPSS software package version 19 (IBM Corp. Armonk, NY, United States). The significance of differences between two or multiple groups was evaluated by Student’s *t*-test and one-way ANOVA. Data were expressed as the mean ± standard deviation (SD) and considered significantly different at *p* < 0.05. The correlation between two variables was tested by Pearson product-moment correlation method and was considered significant at *p* < 0.05.

### Nucleotide Sequence Accession Numbers

The representative bacterial and archaeal *amoA* gene sequences reported in this study have been deposited in GenBank under accession numbers MH316770–MH316814 and MH316815–MH316856.

## Results

### Physicochemical Characteristics of Different Aquaculture Ponds

The water characteristics of the ponds in the different sampling areas are listed in **Supplementary Table S4**. The water temperature and NO_2_^-^ in the Heilongjiang ponds were significantly lower (*p* < 0.05), but pH, DO, and NH_4_^+^ were significantly higher (*p* < 0.05) compared to ponds in other areas. The water pH values in the Heilongjiang ponds ranged from 8.9 to 9.38, indicating an alkaline environment in Heilongjiang pond water.

Despite the water being alkaline, the sediment in the Heilongjiang ponds was relatively neutral, with a pH range from 7.44 to 7.86, and showed no significant difference to ponds in other areas. Sediment TN in different ponds ranged from 1438.40 to 2528.12 mg/kg, TP ranged from 798.43 to 2291.96 mg/kg, NO_3_^-^ ranged from 0.40 to 2.32 mg/kg, NH_4_^+^ ranged from 22.57 to 547.62 mg/kg, DOC ranged from 67.71 to 949.75 mg/kg, urea ranged from 1.45 to 2.90 μg/g, and water- and acid-soluble sulfate ranged from 84.70 to 2188.87 and 0 to 7154.50 mg/kg, respectively. The differences of these parameters in different pond areas were not significant, however sediment NO_2_^-^, TOC, MBC and enzymes showed significant differences among the ponds in different areas (**Table 1**).

**Table 1:** Comparison of sediment characteristics between ponds in different sampling areas^∗^.

Area	NO_2_^-^ (mg/kg)	TOC (g/kg)	MBC (mg/kg)	Glu (mg *p*-nitrophenol kg^-1^ soil h^-1^)	Ure (mg NH_4_^+^ kg^-1^ soil 24 h^-1^)	Ary (mg *p*-nitrophenol kg^-1^ soil h^-1^)
CJ	0.07 ± 0.04^b^	8.33 ± 1.24^b^	286.19 ± 110.61^a,b^	52.00 ± 5.61^b^	663.48 ± 466.22^b^	189.81 ± 134.96^b^
HLJ	0.06 ± 0.01^b^	11.01 ± 1.88^a,b^	132.41 ± 117.45^b^	72.56 ± 6.57^a^	1207.41 ± 586.47^b^	465.12 ± 236.91^a,b^
ZJ	0.23 ± 0.13^a^	12.66 ± 2.31^a^	478.04 ± 243.79^a^	56.98 ± 12.25^ab^	2108.01 ± 147.94^a^	691.23 ± 179.07^a^

^∗^*The average values of ponds in different sampling areas are indicated. Pond samples: HLJ, Heilongjiang; ZJ, Zhujiang; CJ, Changjiang. NO_*2*_^-^-N, nitrite; TOC, total organic carbon; MBC, microbial biomass carbon; Glu, β-glucosidase; Ure, urease; Ary, arylsulfatase. The different letters in the upper right-hand corner (a, b) indicate a significant difference (*p* < 0.05) based on the analysis of variance*.

The nitrite concentrations in Zhujiang pond sediments were significantly higher than in other areas (*p* < 0.05), ranging from 0.21 to 0.36 mg/kg. Sediment TOC and MBC in the Zhujiang ponds were significantly higher compared to the Changjiang and Heilongjiang ponds (*p* < 0.05), respectively, indicating relatively higher organic matter content in Zhujiang pond sediments. β-glucosidase in Heilongjiang pond sediments was significantly higher than in Changjiang ponds (*p* < 0.05), ranging from 67.20 to 79.89 mg *p*-nitrophenol kg^-1^ soil h^-1^, indicating relatively higher C-cycling activities in Heilongjiang pond sediments. Urease was significantly higher in Zhujiang pond sediments compared to ponds in other areas (*p* < 0.05), ranging from 1957.06 to 2252.74 mg NH_4_^+^ kg^-1^ soil 24 h^-1^, which suggests higher rates of nitrogen decomposition in Zhujiang pond sediments. The values of sediment arylsulfatase in the Zhujiang ponds ranged from 576.28 to 897.54 mg *p*-nitrophenol kg^-1^ soil h^-1^, and were significantly higher than in the Changjiang ponds (*p* < 0.05), indicating relatively higher S-cycling activities in Zhujiang pond sediments.

### *amoA* Gene Abundances and Potential Ammonia Oxidation Rates

**FIGURE 1:**
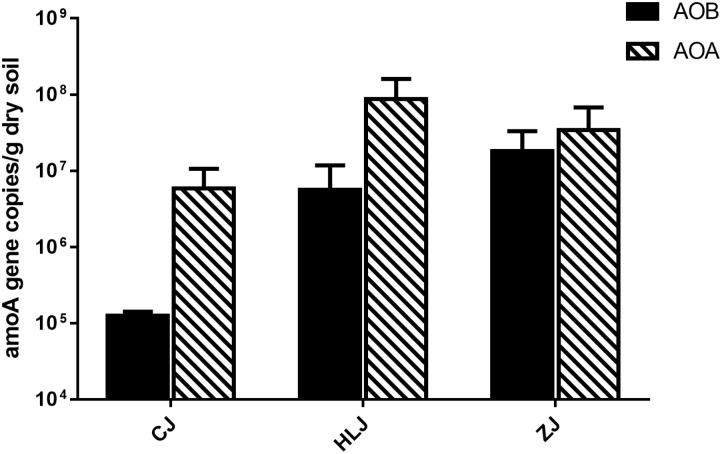
Abundance of bacterial and archaeal *amoA* gene in different sampling areas. Pond samples: CJ, Changjiang; HLJ, Heilongjiang; ZJ, Zhujiang.

The copy number of AOB *amoA* genes ranged from 1.07 × 10^5^ to 2.90 × 10^7^ copies/g dry soil, while AOA ranged from 1.04 × 10^6^ to 1.30 × 10^8^ copies/g dry soil (**Figure 1**). AOA were more abundant than AOB in Changjiang and Heilongjiang ponds. AOA and AOB numbers were not significantly different in Zhujiang pond sediments. The ratios of AOA/AOB *amoA* gene copies ranged from 0.61 to 76.45. Compared to other sampled areas, there were more AOB copies in the Zhujiang ponds and more AOA copies in the Heilongjiang ponds. However, AOB and AOA copies in different sampling areas were not significantly different, possibly due to large variations between different ponds.

The potential ammonia oxidation rates (PNRs) in different sampling ponds were determined to assess possible ammonia oxidation activities in the sediment. PNRs ranged from 6.37 to 63.76 ng NO_2_-N/g dry mass of soil/h, but were not significantly different between ponds in different areas. Correlation analyses indicate significant correlation between the abundance of AOB and arylsulfatase (*R*^2^ = 0.679, *p* < 0.05), as well as the abundance of AOA and TN (*R*^2^ = 0.791, *p* < 0.05) and arylsulfatase (*R*^2^ = 0.716, *p* < 0.05). Additionally, the AOB/AOA ratio was found to be significantly correlated with MBC (*R*^2^ = 0.699, *p* < 0.05). However, no significant correlations were detected between the AOA/AOB abundance and PNRs as well as NO_2_^-^, NO_3_^-^, and NH_4_^+^.

### Phylogeny of Bacterial and Archaeal *amoA* Genes

#### Ammonia-Oxidizing Bacteria (AOB)

**FIGURE 2:**
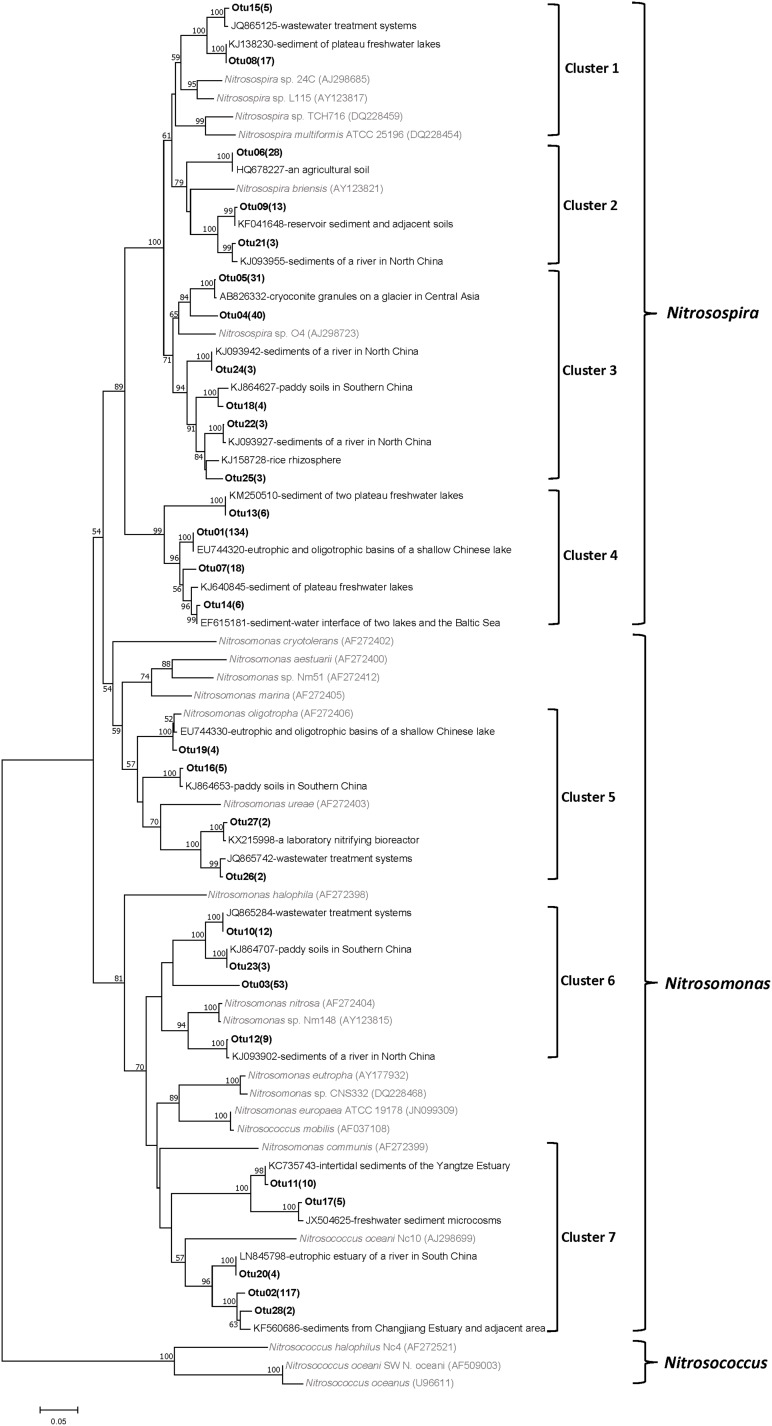
Phylogenetic tree of representative AOB OTU and reference sequences from GenBank. The bold number in parentheses behind OTU represents the numbers of the sequences detected. Numbers at the nodes indicate the levels of bootstrap support based on neighbor-joining analysis of 1000 resampled datasets. Only bootstrap values > 50% are represented. The scale bar represents 5% sequence divergence.

A total of 45 AOB OTUs were detected in all pond sediments based on a 3% divergence cutoff, including 11, 29, and 23 OTUs in the Changjiang, Heilongjiang and Zhujiang ponds, respectively. The representative OTUs were grouped into seven clusters with robust phylogenetic support based on their relative similarity to phylogenetic reference sequences, covering two out of the three main lineages in the AOB phylogeny (**Figure 2**).

Sequences identified as belonging to *Nitrosospira* were grouped into Cluster 1 (85.8% similar to *Nitrosospira multiformis* ATCC 25196), Cluster 2 (88.2–89.2% similar to *Nitrosospira briensis*), Cluster 3 (84.8–86.7% similar to *Nitrosospira briensis*), and Cluster 4 (74.9–78.9% similar to *Nitrosospira briensis*). Sequences grouped into Cluster 1 and Cluster 4 were mainly detected in the Changjiang and Heilongjiang ponds. Sequences grouped into Cluster 2 were detected in the Heilongjiang and Zhujiang ponds. Sequences grouped into Cluster 3 were mainly detected in the Heilongjiang ponds.

Sequences identified as belonging to *Nitrosomonas* were grouped into Cluster 5 (84.2–98.5% similar to *Nitrosomonas oligotropha* or *Nitrosomonas ureae*), Cluster 6 (83.6–90.7% similar to *Nitrosomonas nitrosa*), and Cluster 7 (74.9–82.4% similar to *Nitrosomonas communis*). Sequences grouped into Cluster 5 were detected in the Heilongjiang and Zhujiang ponds. Sequences grouped into Cluster 6 and Cluster 7 were detected in all sampling areas.

#### Ammonia-Oxidizing Archaeal (AOA)

**FIGURE 3:**
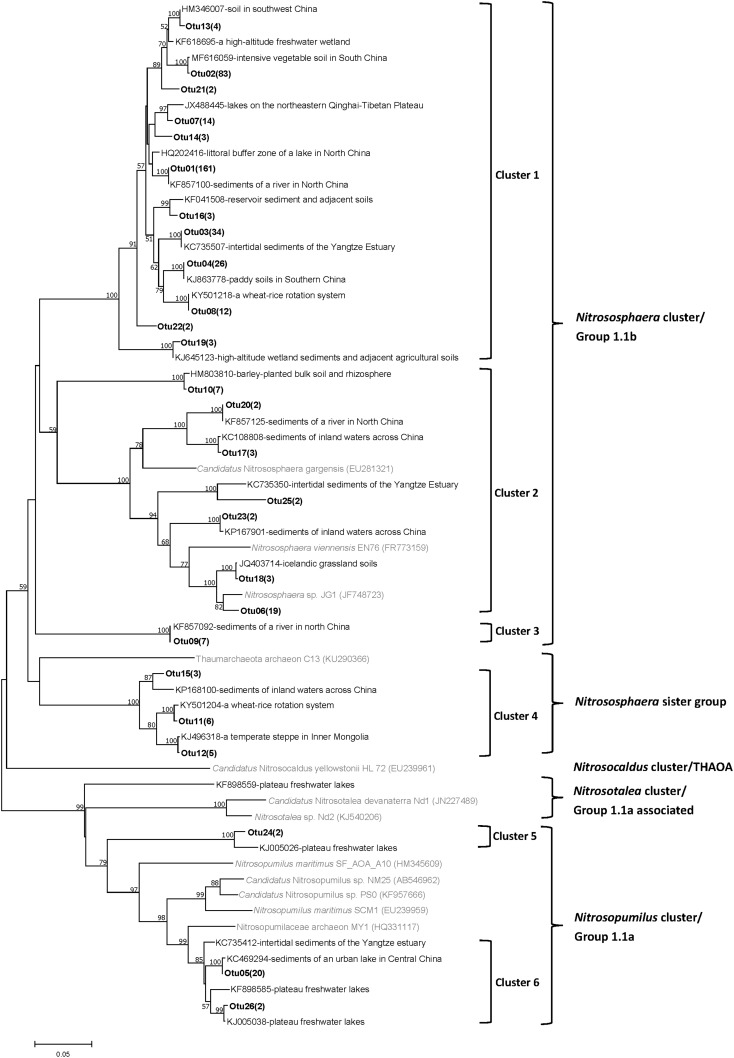
Phylogenetic tree of representative AOA OTU and reference sequences from GenBank. The bold number in parentheses behind OTU represents the numbers of the sequences detected. Numbers at the nodes indicate the levels of bootstrap support based on neighbor-joining analysis of 1000 resampled datasets. Only bootstrap values > 50% are represented. The scale bar represents 5% sequence divergence.

A total of 42 AOA OTUs were detected in all pond sediments based on a 3% divergence cutoff, including 17, 11, and 22 OTUs in the Changjiang, Heilongjiang, and Zhujiang ponds, respectively. The representative OTUs were grouped into six clusters based on their relative similarity to the reference sequences, covering three out of the five main lineages in the AOA phylogeny (**Figure 3**).

Sequences identified as belonging to *Nitrososphaera* cluster/Group 1.1b were grouped into Cluster 1 (74.4–79.3% similar to *Nitrososphaera viennensis* EN76 or *Candidatus* Nitrososphaera gargensis), Cluster 2 (74.2%–91.3% similar to *Nitrososphaera viennensis* EN76 or *Candidatus* Nitrososphaera gargensis), and Cluster 3 (only one OTU, 75% or 77.5% similar to *Nitrososphaera viennensis* EN76 or *Candidatus* Nitrososphaera gargensis, respectively). Sequences grouped into Cluster 3 were only detected in the Zhujiang ponds, and Cluster 1 and Cluster 2 included sequences from all sampling areas.

Cluster 4 fell into a phylogenetic group known as the *Nitrososphaera* sister group which was first proposed in 2011 (Pester et al., 2012). Sequences were grouped with an assembled a*moA* gene sequence (with similarity of 78.1–79.1%) from a novel AOA belonging to the *Nitrososphaera* sister cluster (Lehtovirta-Morley et al., 2016). The sequences in Cluster 4 were detected in the Changjiang and Heilongjiang but not the Zhujiang ponds.

The remaining sequences were identified as *Nitrosopumilus* Cluster/Group 1.1a AOA and were divided into two clusters, Cluster 5 and Cluster 6. Cluster 5 contained only one OTU (78.7% similar to *Nitrosopumilus maritimus* SCM1) and was detected in only the Changjiang and Zhujiang ponds. Cluster 6 sequences showed relatively higher identities to *Nitrosopumilus maritimus* SCM1 (88.6–88.8%), and was distributed in only the Chanjiang and Zhujiang ponds.

### AOB and AOA Diversity and Community Structure

Rarefaction analysis of bacterial and archaeal *amoA* gene libraries (**Supplementary Figure S1**) showed an average coverage of 94 and 91%, respectively, and no significant differences were found in coverage between the different sampling areas.

The average values of AOB richness and the diversity indices of pond sediments in the different sampling areas are presented in **Supplementary Table S5**. The OTU numbers and diversity of AOB were slightly lower in the Changjiang ponds compared to those in other areas, but the difference was not statistically significant. However, the OTU numbers and diversity of AOA were significantly different (*p* < 0.05), particularly between the Heilongjiang and Zhujiang ponds (**Table 2**). The Zhujiang ponds had higher AOA richness and abundance than the Heilongjiang ponds. The values of Chao1 (19–21.5), and Shannon (1.66–2.33) in the Zhujiang ponds indicated a relatively high AOA diversity in Zhujiang pond sediments.

**Table 2:** Richness and diversity indices of AOA OTUs in different pond sediments^∗^.

	sobs	chao	ace	jackknife	shannon	npshannon	simpson
CJ	10^ab^	10.60^b^	13.82^b^	13.00^ab^	1.57^ab^	1.74^ab^	0.28^b^
HLJ	6^b^	8.33^b^	12.36^b^	4.33^b^	0.98^b^	1.18^b^	0.54^a^
ZJ	13^a^	20.00^a^	22.90^a^	29.16^a^	2.09^a^	2.31^a^	0.15^b^

^∗^*The average values of ponds in different sampling areas are indicated. Pond samples: CJ, Changjiang; HLJ, Heilongjiang; ZJ, Zhujiang. “sobs” represents the number of observed OTUs, “chao” represents the values of the Chao1 richness estimator. OTUs were calculated based on 3% sequence variation. The different letters in the upper right-hand corner (a,b) indicate a significant difference (*p* < 0.05) based on the analysis of variance. The diversity indexes were calculated by MOTHUR (v. 1.39.5)*.

Statistical analyses indicated that the AOB Shannon index was significantly correlated with TOC (*R*^2^ = 0.691, *p* < 0.05), PNR (*R*^2^ = 0.740, *p* < 0.05), and arylsulfatase (*R*^2^ = 0.734, *p* < 0.05), while the AOA Shannon index was significantly correlated with MBC (*R*^2^ = 0.673, *p* < 0.05) and β-glucosidase.

**FIGURE 4:**
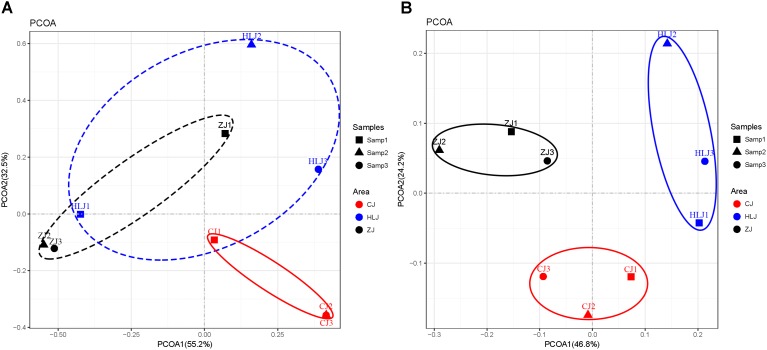
Principal coordinates analysis (PCoA) using weighted UniFrac algorithm of AOB **(A)** and AOA **(B)** communities revealed by *amoA* genes. Pond samples: CJ, Changjiang; HLJ, Heilongjiang; ZJ, Zhujiang.

The representative *amoA* gene sequences were used to make comparisons between the microbial community structures of different sampling areas through principal coordinates analysis (PCoA) based on weighted UniFrac distances (**Figure 4**). The AOB communities of Changjiang pond sediments deviated from those of the Zhujiang and Heilongjiang ponds, as they were distributed along separate areas of the axes with a total explanation percentage of 87.7%. However, the AOB communities of Zhujiang and Heilongjiang pond sediments tended to group together (**Figure 4A**). Clustering analysis using OTU sequences from the pond samples also indicated a similarity between Zhujiang and Heilongjiang AOB communities (**Supplementary Figure S2**), probably due to shared OTUs between the Zhujiang and Heilongjiang samples (a total of 13 OTUs were found to be shared between the two areas).

However, AOA communities from different areas were distinctively separated along the axes with a total explanation percentage of 71% (**Figure 4B**), indicating the presence of distinctive AOA communities in the different sampling areas. Similarly, the AOA OTUs of different areas were also clearly separated by clustering analysis (**Supplementary Figure S2**).

### AOB and AOA Community Compositions and Potential Environmental Drivers

#### AOB and AOA Community Compositions

**FIGURE 5:**
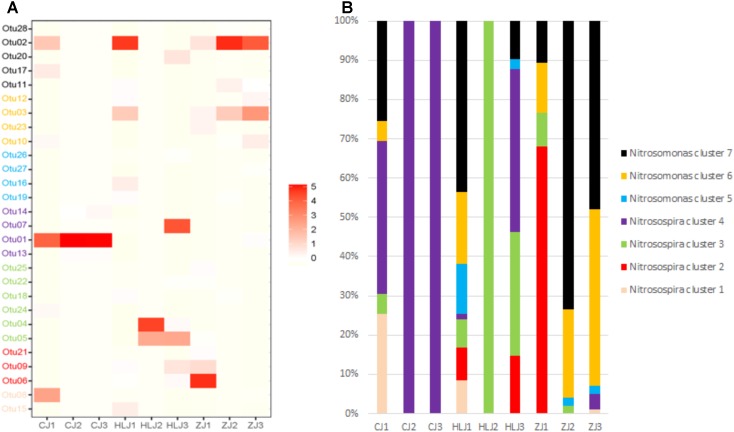
Heatmap showing the relative abundance of AOB representative OTUs among different ponds **(A)** and frequency of each identified AOB cluster/species in different sampling areas **(B)**. The colors in the heatmap represent the lowest to highest frequency of OTUs, from white to red, respectively. Pond samples: CJ, Changjiang; HLJ, Heilongjiang; ZJ, Zhujiang.

The representative AOB OTUs were displayed in a heatmap to show the microbial compositions of pond sediments in the different sampling areas (**Figure 5A**). The identified OTUs of AOB were further referred back to their phylogenetic structure and grouped into species. The proportions of the detected species in each pond sample are shown in **Figure 5B**. In Changjiang pond sediments, *Nitrosospira* cluster 4 was the most prominent AOB cluster/species, mainly in the form of Otu01. *Nitrosomonas* cluster 7 and *Nitrosospira* cluster 3 were the most prominent clusters in Heilongjiang pond sediments, mainly consisting of Otu02, Otu04, and Otu05. *Nitrosospira* cluster 2 and *Nitrosomonas* cluster 7 were the most prominent clusters in Zhujiang ponds, mainly containing Otu06 and Otu02.

**FIGURE 6:**
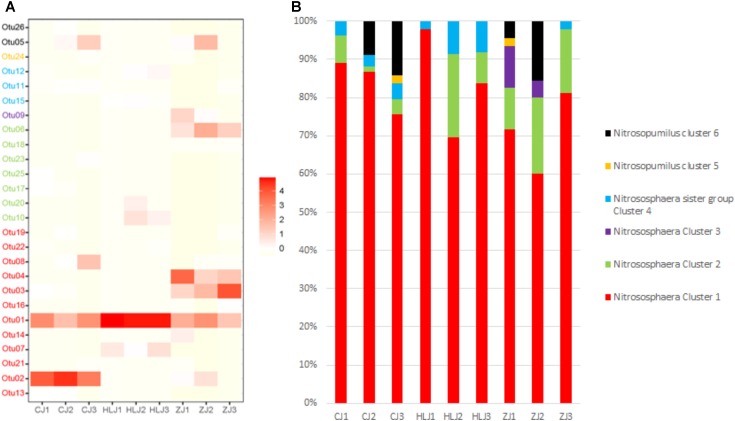
Heatmap showing the relative abundance of AOA representative OTUs among different ponds **(A)** and frequency of each identified AOA cluster/species in different sampling areas **(B)**. The colors in the heatmap represent the lowest to highest frequency of OTUs, from white to red, respectively. Pond samples: CJ, Changjiang; HLJ, Heilongjiang; ZJ, Zhujiang.

For AOA OTUs, the most prominent cluster/species was *Nitrososphera* Cluster 1 for all pond sediments in the different sampling areas (**Figure 6A**). Additionally, *Nitrososphera* Cluster 1 also constituted large percentages of the species compositions (**Figure 6B**). Otu01 and Otu02 were the most prominent AOA cluster/species in Changjiang pond sediments. Otu01 was the most prominent AOA in Heilongjiang pond sediments. For the Zhujiang ponds, in addition to *Nitrososphera* Cluster 1 cluster/species (Otu01, Otu03, and Otu04), Otu06 from *Nitrososphaera* Cluster 2 also contributed significantly to the AOA species composition.

#### Potential Environmental Drivers

**FIGURE 7:**
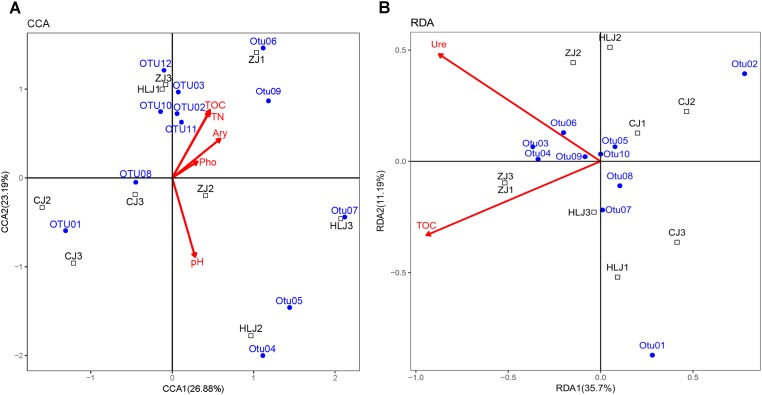
CCA and RDA ordination plots for the relationship between AOB **(A)** and AOA **(B)** prominent OTUs (sequence numbers ≥ 7) with the environmental parameters in different ponds. Pond samples: CJ, Changjiang; HLJ, Heilongjiang; ZJ, Zhujiang. TOC, total organic carbon; TN, total nitrogen; Ary, arylsulfatase; Pho, acid phosphatase; Glu, β-glucosidase; Ure, urease.

The correlations between AOB and AOA community compositions and the associated environmental variables were analyzed by multivariate analysis CCA and RDA, respectively (**Figure 7**). The environmental variables in the first two dimensions of CCA and RDA explained 50.07 and 46.89%, respectively, of the total variance in the bacterial (**Figure 7A**) and archaeal (**Figure 7B**) prominent *amoA* genotype compositions (OTUs with sequence numbers ≥ 7).

Sediment TOC, TN, pH, acid phosphatase and arylsulfatase were significantly correlated with the prominent AOB *amoA* genotypes (**Supplementary Table S6**). These factors provided 26.88, 23.19, 11.19, 5.69, and 0.58% of the total CCA explanatory power. Most OTUs of AOB were positively correlated with sediment TOC, TN, acid phosphatase, and arylsulfatase levels, but negatively correlated with sediment pH.

For AOA, TOC and urease were found to be significantly correlated with the prominent *amoA* genotype compositions (**Supplementary Table S7**), and provided 35.70 and 11.19% of the total RDA explanatory power. Most of the AOA OTUs were positively correlated with TOC, including Otu01, which was detected in all the sampled ponds. However, several of the dominant OTUs were found to be negatively correlated with urease, including Otu01 and Otu02.

## Discussion

### AOB and AOA Abundance in Surface Sediments of Aquaculture Ponds

Our understanding on AOB and AOA abundance in aquaculture pond sediments is still very limited. The predominance of AOB or AOA varies between different freshwater sediments (Yang et al., 2016), and inconsistent results have been found in previous aquaculture environment studies. For example, AOB abundance was found to be higher than AOA in *Mandarin* fish pond sediments (Zhou et al., 2017), while the abundance of AOA was nearly an order of magnitude higher than AOB in polyculture pond sediments (Lu et al., 2015, 2016). In this study, AOA were predominant in all the aquaculture ponds except one, but great variations in AOB and AOA abundance were found between different ponds. Although a much higher abundance of AOB (up to 2.90 × 10^7^ copies/g dry soil) and AOA (up to 1.30 × 10^8^ copies/g dry soil) were detected in some of our aquaculture ponds compared to the aforementioned studies, or even compared to some soils (Wang J. et al., 2014; Tago et al., 2015), the abundance of AOB and AOA was up to two orders of magnitude lower in other ponds. Considering the significant differences between aquaculture ponds (**Table 1**), the large variations in terms of abundance could be due to the different trophic statuses.

Due to the large amounts of decomposition during culturing resulting from feeding debris and species excrements, the surface sediments of aquaculture ponds are usually rich in organic substances and may exhibit high NH_4_^+^ concentrations. Ammonia concentration is considered an important factor that might affect the abundance of AOB/AOA *amoA* genes, due to their different affinities to NH_4_^+^ (Martens-Habbena et al., 2009). However, our results indicated that the abundance of AOA/AOB was not significantly correlated with NH_4_^+^. By comparing our results with those of other studies, we found that the concentrations of NH_4_^+^ in our study (up to 547.62 mg/kg) were much higher than in other environments (lower than 50 mg/kg, like soils e.g., Wang et al., 2015; and sediments e.g., Zheng et al., 2013). Such high concentrations may indicate that NH_4_^+^ might not be a limiting factor for AOB/AOA in aquaculture pond sediments, which correlates with similar results found in a previous study on aquaculture ponds (Lu et al., 2016). In fact, lower concentrations of NH_4_^+^ (28.40–72.93 mg/kg) have been found to be more than what is required to support AOA communities in forest soils (Wu et al., 2017). Thus, we deduced that NH_4_^+^ might not be a significant regulating factor for AOB/AOA in aquaculture pond sediments with high NH_4_^+^ concentrations. Organic substances, on the other hand, are another important indicator of trophic state and have been shown to greatly affect AOB/AOA abundance in trophic freshwater sediments (e.g., Herrmann et al., 2009; Wu et al., 2010; Hou et al., 2013). Due to the possible inhibition of AOA by organic compounds (Könneke et al., 2005), AOB tended to outnumber AOA in organic-rich environments, as illustrated by the negative correlation between the abundance of AOA and TOC (e.g., Wu et al., 2010). In contrast, a positive correlation between AOA abundance and TOC was found in an aquaculture pond (Lu et al., 2016), indicating that very different conditions might exist in aquaculture pond sediments, however, the mechanisms of which are unknown. In the present study, we used sediment enzymes to evaluate the dynamics of organic substances in aquaculture pond sediments. Similarly, AOA/AOB abundance was found to be positively correlated with TOC in our study. A significantly positive correlation was found between the abundance of AOB and the concentration of arylsulfatase (*R*^2^ = 0.679, *p* < 0.05), as well as the abundance of AOA and TN (*R*^2^ = 0.791, *p* < 0.05) and arylsulfatase (*R*^2^ = 0.716, *p* < 0.05). To our knowledge, this is the first study to report correlations between arylsulfatase in sediment and ammonia-oxidizing microorganisms in freshwater ecosystems.

Enzymes, suggested to be good indicators of soil quality because of their important role in the decomposition of organic matter as well as in nutrient cycling (Aon et al., 2001), are commonly analyzed components of in soil (e.g., Li and Sarah, 2003; Bowles et al., 2014). Air-drying is an important practice in soil characterization because it allows for easy handling (Zornoza et al., 2006; Lopes et al., 2015) and could possibly eliminate the activity of unstable enzymes and leave only stable enzymes, which could have an important ecological effect on soil quality (Nannipieri et al., 1990). However, the evaluation of enzymes in sediments is still very limited. In this study, air-dried sediments were used to evaluate four enzymes which have been proved stable and important in nutrient cycling (Bandick and Dick, 1999; Li and Sarah, 2003). Among these, arylsulfatase catalyzes the hydrolysis of organic sulfate esters and is primarily produced by sediment microorganisms, and may constitute a rate-limiting stage in S cycling (Tabatabai and Bremner, 1970; Chröst, 1991). In this study, arylsulfatase was analyzed to evaluate the process of S-related organic nutrients, considering the high input of protein-rich feed which release S after being decomposed. Indeed, sulfate, as a decomposition product of arylsulfatase, could be detected in the sediments of our aquaculture ponds. Thus, the correlation of AOA and AOB abundance to arylsulfatase may indicate a relationship between ammonia oxidizers and the availability of organic sulfur compounds to microorganisms in aquaculture pond sediments. We further deduced that organic matter (especially organic sulfur) related microorganisms may contribute to the positive correlation between AOB/AOA abundance and TOC in aquaculture pond sediments. Indeed, our results also showed a significant correlation between AOB/AOA ratio and MBC, indicating a relationship between AOB/AOA and sediment microorganisms. However, limited information is known about the impact of organic substance dynamics on microorganisms as well as their interconnections in aquaculture pond sediments. Further studies are needed to elucidate the contribution of TOC on the abundance of ammonia-oxidizing microorganisms in aquaculture ponds.

### Potential Ammonia Oxidation Rate in Surface Sediments of Aquaculture Ponds

Considering the unnecessary correlation between the *amoA* gene and the activity of ammonia oxidation in organisms (Prosser and Nicol, 2008; Veuger et al., 2013), ammonia oxidation rate has usually been determined to obtain more comprehensive information. Many studies use the ^15^NH_4_^+^ isotope dilution technique to detect ammonia oxidation rates in water (Ward, 2011) and sediment (Chen et al., 2016). The addition of chlorate to soil slurry is another method for determining ammonium oxidation, which is suggested by the International Organization for Standardization [ISO 15685:2012(E)]. The chlorate inhibition method has been successfully applied in the past to determine potential ammonia oxidation in soil (e.g., Kurola et al., 2005; Hu et al., 2015; Hayashi et al., 2016) and sediment environments (e.g., Hall, 1984; Kitidis et al., 2011; Laverock et al., 2013). In addition, a recent study on aquaculture pond sediments also used this method to determine the potential ammonia oxidation rates (Lu et al., 2016). Thus, we deduced that the method for determining potential ammonia oxidation rates using chlorate inhibition in our study could produce reliable and comparable results. Indeed, the PNRs detected in this study correlated with those of the above-mentioned studies, that is relatively lower than detected in the soils (Kurola et al., 2005; Hu et al., 2015), but higher than in the sediment of an aquaculture pond (Lu et al., 2016). In addition, a relatively smaller range of PNRs was detected in our study compared to those in soils, possibly indicating ubiquitous inhibition on the activity of nitrifiers in aquaculture pond sediments, especially considering the similarity of low PNRs between pond samples of different areas.

### AOB and AOA Diversity in Surface Sediments of Aquaculture Ponds

Like with abundance, there are few reports regarding AOB and AOA diversity in aquaculture pond sediments. Zhou et al. (2017) reported higher AOB diversity than AOA in the sediment of ponds with *Mandarin* fish. In the present study, different results were observed between ponds. AOA OTU richness and diversity were higher than AOB in the sediments of Changjiang and Zhujiang ponds, but AOB was higher in Heilongjiang pond sediments (see **Table 2** and **Supplementary Table S5**), indicating varied AOB/AOA diversity in different aquaculture pond sediments. The eutrophic level of freshwater sediments have been suggested to positively influence ammonia-oxidizing microbial populations (Yang et al., 2016). Correlation analysis of different ponds in our study also suggested that sediment AOB diversity was positively affected by TOC (*R*^2^ = 0.691, *p* < 0.05). Moreover, AOB and AOA diversity were also significantly correlated with arylsulfatase (*R*^2^ = 0.734, *p* < 0.05) and β-glucosidase (*R*^2^ = 0.673, *p* < 0.05), respectively. These are important extracellular enzymes during transformation of organic matter, and are primarily produced by microbial organisms in sediment. As such, the activity of microorganisms decomposing organic matter might influence ammonia oxidizers in aquaculture pond sediments. Indeed, AOA diversity was also significantly correlated with MBC in our sampling of pond sediments.

### AOB and AOA Community Structure in Surface Sediments of Aquaculture Ponds

The archaeal and bacterial *amoA* gene sequences in this study were found to be closely related with those detected in soil and freshwater environments (**Figures 2**, **3**), indicating a high homology of ammonia oxidizers in aquaculture pond sediments and other environments. All AOB were affiliated to *Nitrosospira* and *Nitrosomonas* clusters, similar to previous studies in aquaculture pond sediments (Lu et al., 2016) and freshwater lake sediments (Bao et al., 2016). However, there were also large variations between different AOB OTUs (similarity to known isolates varied from 74.9 to 98.5%), and different AOB clusters were distributed in our sampling areas, indicating large variations in the AOB community in aquaculture pond sediment. Similarly, different AOA community compositions were also detected in the sampling areas. AOA were classified into the *Nitrososphaera* cluster, *Nitrososphaera* sister cluster, and *Nitrosopumilus* cluster according to Pester et al. (2012), which was in accordance with studies on freshwater sediments (Zheng et al., 2013; Yang et al., 2016), but large variations were found between different clusters (similarity to known isolates varied from 74.2 to 91.3%). Pond sediments in different areas were also distributed with varying AOA clusters.

PCoA analysis with representative *amoA* gene sequences further identified AOB and AOA community variations in the pond sediments of different sampling areas (**Figure 4**). Although the levels of sediment AOB showed some similarity between the Heilongjiang and Zhujiang ponds, most ponds in these areas were distinctly different. Indeed, sediment AOB communities in these areas were dominated by different *amoA* gene sequences (**Figure 5A**), and different AOB phylogenetic clusters were detected in the sampling areas (**Figure 5B**). The proportions of *Nitrosospira* and *Nitrosomonas* were found to be varied in lake sediments (Liu et al., 2015). In aquaculture environments, both *Nitrosospira* and *Nitrosomonas* could be predominant (Lu et al., 2016; Zhou et al., 2017). In the present study, the *Nitrosospira* cluster was prominent in the Changjiang ponds, while the *Nitrosomonas* and *Nitrosospira* clusters were prominent in the Heilongjiang and Zhujiang ponds. This suggests that niche separation might exist for AOB species in aquaculture pond sediments. Previous studies on freshwater sediments have indicated that AOB components were differently distributed along the trophic gradients from mesotrophic to eutrophic (Hou et al., 2013; Yang et al., 2016). In this study, AOB were also found to be significantly influenced by sediment eutrophic status (TOC and TN levels), and pH (**Figure 7A**). In addition, acid phosphatase and arylsulfatase in sediments were significantly correlated with AOB prominent *amoA* genotypes, indicating that the bio-availability of organic P and S might significantly affect AOB communities in aquaculture pond sediments.

For AOA, the *Nitrososphaera* cluster was dominant in all the sampling pond sediments (**Figure 6**), a similar result seen in previous studies on aquaculture pond sediments (Lu et al., 2016; Zhou et al., 2017), but more AOA clusters were found in our study (e.g., the *Nitrosopumilus* Cluster). *Nitrososphaera* AOA were found to be dominant in mesotrophic lakes compared to oligotrophic lakes (Bollmann et al., 2014; Yang et al., 2016). The dominance of the *Nitrososphaera* cluster in our ponds may be due to the high eutrophic levels of aquaculture pond sediments compared to those of other environments. In addition, although the predominance of *Nitrososphaera* cluster was ubiquitous, the sediment AOA species and phylogenetic compositions varied in different sampling areas. RDA analysis using representative archaeal *amoA* gene sequences indicated that TOC significantly contributed to the variety in AOA communities observed in different areas. However, dominant *Nitrososphaera* cluster sequences (Otu01 and Otu02) were found to be negatively correlated with urease, which is important in N-cycling in sediments, indicating a negative effect of nitrogen transformation on *Nitrososphaera* cluster species in aquaculture ponds. Previous study on the soils of different geographic regions have also indicated the negative effect of soil nitrogen on the AOA *Nitrososphaera* cluster (Pester et al., 2012). However, further ecophysiological studies based on cultivation are needed to better understand the ecology of these ammonia oxidizers.

## Conclusion

In conclusion, this study revealed that AOB and AOA were abundant in *Ctenopharyngodon idellus* aquaculture pond sediments. The predominance of AOB or AOA varied between the sediments of different aquaculture ponds according to the levels of TOC. In particular, the abundance of AOB and AOA was significantly correlated with arylsulfatase, indicating a relationship between ammonia oxidizers and the availability of organic sulfur compounds in the surface sediment of aquaculture ponds. AOB and AOA diversity were also significantly correlated with arylsulfatase and β-glucosidase, respectively, in aquaculture pond sediments, indicating a correlation between AOB and AOA diversity and the activity of microorganisms decomposing organic matter. In addition, the AOA and AOB community structures were significantly correlated with TOC. Taken together, our results suggest that AOB and AOA communities in the surface sediments of *Ctenopharyngodon idellus* aquaculture ponds were regulated by the organic matter found in the ponds, as well as its availability to the microorganisms.

## Author Contributions

XL, LT, and GL performed the design of the initial studies. LD designed the experiments and wrote the draft manuscript. LY revised the manuscript. CS and LP performed the sampling work. LD and CL performed the laboratory work. All the authors have read and approved the final draft of the article.

## Conflict of Interest Statement

The authors declare that the research was conducted in the absence of any commercial or financial relationships that could be construed as a potential conflict of interest.

## Footnotes

1 http://www.ncbi.nlm.nih.gov
