# Supplementary material for: Organic Matter Regulates Ammonia-Oxidizing Bacterial and Archaeal Communities in the Surface Sediments of Ctenopharyngodon idellus Aquaculture Ponds
Source: Front Microbiol. 2018 Sep 24;9:2290. doi: 10.3389/fmicb.2018.02290 (PMC6165866; doi:10.3389/fmicb.2018.02290)
Supplement: Supplementary file 1 [file Table_1.docx]

Supplementary Material

**Organic Matter Regulates** **Ammonia-Oxidizing Bacterial and Archaeal Communities in** **the Surface Sediments of *Ctenopharyngodon idellus* Aquaculture Ponds**

**Lili Dai^1,2^, Chengqing Liu^1,3^, Liqin Yu^2^, Chaofeng Song^1^, Liang Peng^1^, Xiaoli Li^1^, Ling Tao^1^, Gu Li^1*^**

*^*^ Correspondence: Gu Li, ligu@yfi.ac.cn*

**1 Supplementary Figures**

(A)

(B)

**FIGURE S1**. Rarefaction curves of bacterial (a) and archaeal (b) *amoA* genes of ponds in different sampling areas. Pond samples: CJ, Changjiang; HLJ, Heilongjiang; ZJ, Zhujiang. Difference at 3% sequence variation was used to determine the operation taxonomic unit (OTU).

(A) (B)

**FIGURE S2**. Newick-formatted clustering tree based on the Jaccard values of ammonia-oxidizing bacterial (A) and archaeal (B) OTU sequences in pond sediments of different areas. Pond samples: CJ, Changjiang; HLJ, Heilongjiang; ZJ, Zhujiang. The clustering analyses were performed in MOTHUR (v. 1.39.5) and the tree results were opened in TreeViewX.

**2 Supplementary Tables**

**TABLE S1** *Ctenopharyngodon idellus* culturing conditions in three sampling areas

| Aquaculture area | Location | Total area  (hm^2^) | Fingerling size (kg/fish) | Stocking density (kg/hm^2^) | Yield  (kg/hm^2^) |
| --- | --- | --- | --- | --- | --- |
| Changjiang | Jingzhou, Hubei | 8 | 1 | 7,500 | 24,000 |
| Heilongjiang | Suihua, Heilongjiang | 26 | 0.5 | 9,750 | 60,000 |
| Zhujiang | Zhongshan, Guangdong | 45 | 0.25 | 6,000 | 75,000 |

**TABLE S2** PCR amplification primers and reaction conditions

| Microbe/  amplified DNA | Primer name | Sequence (5’-3’) | Amplification condition | Reference^*^ |
| --- | --- | --- | --- | --- |
| Ammonia-oxidizing bacteria (AOB) | amoA-1F | GGGGTTTCTACTGGTGGT | 95 °C for 30 s; 35 cycles of 95 °C for 5 s, 58 °C for 40 s, 72 °C for 70 s; and 80 °C for 20 s | Rotthauwe et al., 1997 |
|  | amoA-2R | CCCCTCKGSAAAGCCTTCTTC |  |  |
| Ammonia-oxidizing archaea (AOA) | Arch-amoAF | STAATGGTCTGGCTTAGACG | 95 °C for 5 min; 32 cycles of 95 °C for 45 s, 58 °C for 45 s, 72 °C for 1 min; and 72 °C for 10 min | Francis et al., 2005 |
|  | Arch-amoAR | GCGGCCATCCATCTGTATGT |  |  |

^*^Rotthauwe, J.H., Witzel, K.P., and Liesack, W. (1997). The ammonia monooxygenase structural gene amoA as a functional marker: molecular fine-scale analysis of natural ammonia-oxidizing populations. *Appl. Environ. Microbiol*. 63, 4704-4712

Francis, C.A., Roberts, K.J., Beman, J.M., Santoro, A.E., and Oakley, B.B. (2005). Ubiquity and diversity of ammonia-oxidizing archaea in water columns and sediments of the ocean. *Proc. Natl. Acad. Sci. U.S.A*. 102, 14683-14688. doi: 10.1073/pnas.0506625102

**TABLE S3** qPCR amplification conditions for bacterial and archaeal *amoA* gene

| Microbe/  amplified DNA | Primer name | Amplification condition | Amplification efficiency | R^2^ |
| --- | --- | --- | --- | --- |
| Ammonia-oxidizing bacteria (AOB) | amoA-1F | 95 °C for 3 min; 45 cycles of 95 °C for 15 s, 57 °C for 20 s, 72 °C for 30 s | 90.2% | 0.9981 |
|  | amoA-2R |  |  |  |
| Ammonia-oxidizing archaea (AOA) | Arch-amoAF | 95 °C for 5 min; 45 cycles of 95 °C for 30 s, 53 °C for 38 s, 72 °C for 45 s | 92.9% | 0.9993 |
|  | Arch-amoAR |  |  |  |

**TABLE S4** Water characteristics of ponds in different sampling areas^*^

| Area | Temp (°C) | pH | DO (mg/L) | TP (mg/L) | NO_2_^-^ (mg/L) | NO_3_^-^ (mg/L) | NH_4_^+^ (mg/L) | TN (mg/L) | Chla (μg/L) |
| --- | --- | --- | --- | --- | --- | --- | --- | --- | --- |
| CJ | 27.2±0.75^a^ | 7.94±0.08^b^ | 6.36±0.75^b^ | 0.80±0.05^a^ | 0.03±0.00^c^ | 0.28±0.06^b^ | 0.68±0.20^b^ | 1.97±0.13^b^ | 39.99±5.81^c^ |
| HLJ | 22.6±1.19^b^ | 9.22±0.27^a^ | 10.37±2.43^a^ | 1.31±0.21^a^ | 0.01±0.00^b^ | 0.11±0.03^b^ | 3.35±1.47^a^ | 12.19±0.46^a^ | 244.24±88.65^b^ |
| ZJ | 28.4±0.26^a^ | 7.63±0.30^b^ | 4.16±1.52^b^ | 0.68±1.75^a^ | 0.50±0.15^a^ | 6.54±1.45^a^ | 0.22±0.20^b^ | 9.71±3.31^a^ | 447.33±65.27^a^ |

^*^The average values of ponds in different sampling areas were indicated. Pond samples: CJ, Changjiang; HLJ, Heilongjiang; ZJ, Zhujiang. Temp, water temperature; DO, dissolved oxygen; TP, total phosphorus; NO_2_^-^, nitrite; NO_3_^-^, nitrate; NH_4_^+^, ammonia nitrogen; TN, total nitrogen; and Chla, chlorophyll a. The different letters in the upper right-hand corner (a, b) indicate a significant difference (*p* < 0.05) based on the analysis of variance.

**TABLE S5** Diversity indexes of ammonia-oxidizing bacterial (AOB) OTUs in different pond sediments^*^

|  | sobs | chao | ace | jackknife | shannon | npshannon | simpson |
| --- | --- | --- | --- | --- | --- | --- | --- |
| CJ | 5 | 5.33 | 5.74 | 1.00 | 0.83 | 0.89 | 0.61 |
| HLJ | 12 | 21.50 | 26.67 | 579.95 | 1.67 | 1.87 | 0.29 |
| ZJ | 10 | 13.92 | 21.78 | 11.08 | 1.51 | 1.68 | 0.33 |

^*^The average values of ponds in different sampling areas were indicated. Pond samples: CJ, Changjiang; HLJ, Heilongjiang; ZJ, Zhujiang. “sobs” represents the number of observed OTUs, “chao” represents the values of the Chao1 richness estimator. OTUs were calculated based on 3% sequence variation. The diversity indexes were calculated by MOTHUR (v. 1.39.5).

**TABLE S6** Permutation tests for the associations between AOB prominent OTUs (sequence numbers ≥ 7) and environmental variables using the function “envfit” of the vegan package in R.

|  | NO_2_^-^ | NO_3_^-^ | NH_4_^+^ | TP | TOC | TN | pH | MBC | DOC | Glu | Pho | Ure | Ary |
| --- | --- | --- | --- | --- | --- | --- | --- | --- | --- | --- | --- | --- | --- |
| r^2^ | 0.527 | 0.271 | 0.443 | 0.621 | 0.780 | 0.843 | 0.783 | 0.338 | 0.555 | 0.600 | 0.606 | 0.621 | 0.728 |
| P value | 0.109 | 0.382 | 0.165 | 0.078 | 0.008^**^ | 0.012^*^ | 0.024^*^ | 0.289 | 0.074 | 0.069 | 0.033^*^ | 0.062 | 0.012^*^ |

NO_2_^-^, nitrite; NO_3_^-^, nitrate; NH_4_^+^, ammonia nitrogen; TP, total phosphorus; TOC, total organic carbon; TN, total nitrogen; MBC, microbial biomass carbon; DOC, dissolved organic carbon; Glu, β-glucosidase; Pho, acid phosphatase; Ure, urease; and Ary, arylsulfatase. Symbols “*” and “**” indicate significant differences at *p* < 0.05 and *p* < 0.01, respectively.

**TABLE S7** Permutation tests for the associations between AOA prominent OTUs (sequence numbers ≥ 7) and environmental variables using the function “envfit” of the vegan package in R

|  | NO_2_^-^ | NO_3_^-^ | NH_4_^+^ | TP | TOC | TN | pH | MBC | DOC | Glu | Pho | Ure | Ary |
| --- | --- | --- | --- | --- | --- | --- | --- | --- | --- | --- | --- | --- | --- |
| r^2^ | 0.549 | 0.168 | 0.269 | 0.568 | 0.751 | 0.434 | 0.351 | 0.611 | 0.595 | 0.580 | 0.470 | 0.745 | 0.520 |
| P value | 0.084 | 0.603 | 0.448 | 0.080 | 0.008^**^ | 0.187 | 0.280 | 0.062 | 0.070 | 0.072 | 0.143 | 0.016* | 0.127 |

NO_2_^-^, nitrite; NO_3_^-^, nitrate; NH_4_^+^, ammonia nitrogen; TP, total phosphorus; TOC, total organic carbon; TN, total nitrogen; MBC, microbial biomass carbon; DOC, dissolved organic carbon; Glu, β-glucosidase; Pho, acid phosphatase; Ure, urease; and Ary, arylsulfatase. Symbols “*” and “**” indicate significant differences at *p* < 0.05 and *p* < 0.01, respectively.
